# GAT-LI: a graph attention network based learning and interpreting method for functional brain network classification

**DOI:** 10.1186/s12859-021-04295-1

**Published:** 2021-07-22

**Authors:** Jinlong Hu, Lijie Cao, Tenghui Li, Shoubin Dong, Ping Li

**Affiliations:** 1 Guangdong Key Lab of Communication and Computer Network, School of Computer Science and Engineering, South China University of Technology, Guangzhou, China; 2 Zhongshan Institute of Modern Industrial Technology, South China University of Technology, Zhongshan, China; 3 Department of Chinese and Bilingual Studies, Faculty of Humanities, The Hong Kong Polytechnic University, Hong Kong, China

**Keywords:** Graph attention networks, Functional brain networks, Resting-state functional connectivity data, Classification, Model interpretation

## Abstract

**Background:**

Autism spectrum disorders (ASD) imply a spectrum of symptoms rather than a single phenotype. ASD could affect brain connectivity at different degree based on the severity of the symptom. Given their excellent learning capability, graph neural networks (GNN) methods have recently been used to uncover functional connectivity patterns and biological mechanisms in neuropsychiatric disorders, such as ASD. However, there remain challenges to develop an accurate GNN learning model and understand how specific decisions of these graph models are made in brain network analysis.

**Results:**

In this paper, we propose a graph attention network based learning and interpreting method, namely GAT-LI, which learns to classify functional brain networks of ASD individuals versus healthy controls (HC), and interprets the learned graph model with feature importance. Specifically, GAT-LI includes a graph learning stage and an interpreting stage. First, in the graph learning stage, a new graph attention network model, namely GAT2, uses graph attention layers to learn the node representation, and a novel attention pooling layer to obtain the graph representation for functional brain network classification. We experimentally compared GAT2 model’s performance on the ABIDE I database from 1035 subjects against the classification performances of other well-known models, and the results showed that the GAT2 model achieved the best classification performance. We experimentally compared the influence of different construction methods of brain networks in GAT2 model. We also used a larger synthetic graph dataset with 4000 samples to validate the utility and power of GAT2 model. Second, in the interpreting stage, we used GNNExplainer to interpret learned GAT2 model with feature importance. We experimentally compared GNNExplainer with two well-known interpretation methods including Saliency Map and DeepLIFT to interpret the learned model, and the results showed GNNExplainer achieved the best interpretation performance. We further used the interpretation method to identify the features that contributed most in classifying ASD versus HC.

**Conclusion:**

We propose a two-stage learning and interpreting method GAT-LI to classify functional brain networks and interpret the feature importance in the graph model. The method should also be useful in the classification and interpretation tasks for graph data from other biomedical scenarios.

## Background

Autism spectrum disorders (ASD) is a spectrum disorder, which means that the symptoms are expressed along a spectrum rather than in a fixed single phenotype. Brain functional connectivity of ASD individuals could be affected at different degree based on the severity of the symptom. Functional connectivity is the statistical relationship between functional brain activities in voxels or regions of interests (ROIs), and it has been used to uncover the complex biological mechanisms in not only typically developing individuals but also neuropsychiatric disorders such as ASD. Given the excellent learning capability, deep learning methods have been used to examine and analyze functional connectivity [1–5]. Functional connectivity vectors are usually used as input data for deep learning models in classifying different phenotypes such as ASD versus healthy controls (HC) [2–7]. To further explore how specific decisions of these networks are made, some explanatory methods, such as piecewise linear neural networks [5], and Shapley value explanation [7], have recently been developed for deep learning models.

Graph neural networks (GNN) have become useful in brain network analyses [8–12]. Unlike standard neural networks using vectors as input data, GNN is a class of Neural Networks for graph data, which retains a state that can represent information of any depth from its neighborhood, and could explore the interactions between graph nodes [13, 14]. GNN has great potential for improving the performance in classifying brain networks. For example, Ktena et al. [8] constructed brain networks based on functional Magnetic Resonance Imaging (fMRI) data, and proposed a Siamese graph convolutional neural network to learn graph similarities for classification. Ma et al. [9] applied similarity learning for brain connectivity networks, and further adopt a random walk strategy with sliding windows to capture the higher-order information of graphs to improve the classification performance. Zhang et al. [10] presented a multi-view graph convolutional network for classifying Parkinson’s Disease cases from controls, where the graph convolutional networks (GCNs), a class of GNN, was applied to extract features from brain networks, and integrated Electronic Health Records with GCN based features for classification. Arslan et al. [11] trained a GCN model for gender classification with brain networks as input, where the global average pooling was used as graph pooling method in the graph model. Gopinath et al. [15] proposed a learnable pooling strategy in GCNs for brain surface analysis, where the neural networks were split to two separate paths, including computing latent features for each node and predicting the node clusters. Finally, Yang et al. [12] developed an edge-weighted graph attention network (GAT) with brain networks as input for classifying Bipolar Disorder, where the dense hierarchical pooling (DHP) [16] was used in the model. These studies attest to the utility and power of GNN and related models.

GAT follows a self-attention strategy and calculates the representation of each node in the graph by attending to its neighbors, and it further uses the multi-head attention [17] to increase the representation capability of the model [14]. To interpret GNN models, a few explanation methods have been applied to GNN classification models. For example, class activation mapping has been used to identify salient nodes (brain regions) [11], and to visualize effective features by gradient sensitivity [12]. These approaches have led to useful insights into the applications of graph neural networks for brain network analysis.

However, it is still challenging to construct accurate graph neural networks and to interpret the specific decisions of these networks for brain network analysis. For example, the pooling method on brain networks is challenging to perform and has room for improvement. In particular, pooling operations for graphs are used to scale down the size of graph representations, and thus reduce overfitting for GNN models [18]. Most pooling methods, such as max-pooling, average-pooling, and DHP, usually follow artificial rules to summarize graph representation from node representation, which would limit the representation ability of the graph. There are also serious challenges to interpret GNN models, as the interpretation of GNNs need to leverage rich relational information and node features in the brain network data.

In this paper, we propose a new graph attention network based learning and interpreting method, namely GAT-LI, which is an accurate graph attention network model for learning to classify functional brain networks, and it interprets the learned graph model with feature importance. Specifically, GAT-LI includes two stages of learning and interpreting. First, in the learning stage, a graph attention network model, namely GAT2, learns to classify functional brain networks of ASD individuals versus healthy controls (HC). In GAT2 model, graph attention layers are used to learn the node representation, and a novel attention pooling layer is designed to obtain the functional brain network representation based on the node representation. Different from artificial rules, the proposed pooling method uses learnable parameters to summarize graph representation from every node’s representation with a unitary learnable standard. Second, in the interpreting stage, we use GNNExplainer [19] to interpret learned GAT2 model with feature importance. GNNExplainer is a model-agnostic approach, which could generate consistent and concise interpretation for an entire class of instances.

We experimentally compared the GAT2 model’s performance against the performances of well-known classification models including support vector machine (SVM), random forest (RF), MultiLayer Perceptron (MLP), convolutional neural networks (CNN), GCN layers based GNN models, and GAT layers based on GNN models in a large dataset containing 1035 subjects from the Autism Brain Imaging Data Exchange I (ABIDE I) database [20]. The results showed that the proposed GAT2 model achieved the highest classification performance. We also experimentally compared the influence of different construction methods of brain networks in the GAT2 model. To further demonstrate the utility and power of GAT2 model, we also experimentally validated the GAT2 model in a larger synthetic graph dataset including 4000 samples.

Finally, we experimentally compared GNNExplainer with two well-known interpretation methods, Saliency Map [21] and DeepLIFT [22], using feature perturbation to interpret the trained GAT2 model. The results showed that the GNNExplainer method interpreted the GAT2 model the best. We further used GNNExplainer to identify the features that have contributed most in classifying ASD cases from healthy controls.

## Methods

In this section, we introduce the construction of functional brain networks, GAT-LI method including GAT2 model and interpretation method, and then we verify the proposed method through classification and interpretation experiments.

### Construction of functional brain networks

**Fig. 1:**
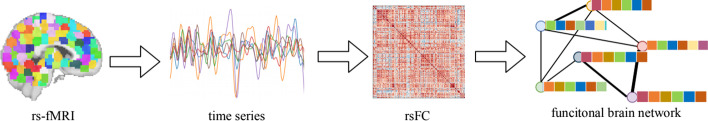
Flow chart of functional brain network construction

The process of functional brain network construction from resting-state fMRI data is shown in Fig. 1.

*Node of network* The whole brain is parcellated into N *ROIs* using the brain atlas. Therefore, each network has N nodes. We use the Harvard Oxford (HO) atlas [23], so we have N = 110 nodes.

*Edge and connectivity matrix* The mean time series of each ROI are extracted, and the resting-state functional connectivity (rsFC) between ROIs are measured by computing the Pearson’s correlation coefficient of the extracted time-series. A $\mathrm{N}\times \mathrm{N}$ connectivity matrix is constructed for each subject respectively, which can be represented as

1 $$S=\left[\begin{array}{cc}\begin{array}{cc}{\rho }_{{r}_{1},{r}_{1}}& {\rho }_{{r}_{1},{r}_{2}}\\ {\rho }_{{r}_{2},{r}_{1}}& {\rho }_{{r}_{2},{r}_{2}}\end{array}& \begin{array}{cc}\cdots & {\rho }_{{r}_{1},{r}_{N}}\\ \cdots & {\rho }_{2,{r}_{N}}\end{array}\\ \begin{array}{cc}\vdots & \vdots \\ {\rho }_{{r}_{N},{r}_{1}}& {\rho }_{{r}_{N},{r}_{2}}\end{array}& \begin{array}{cc}\vdots & \vdots \\ \cdots & {\rho }_{{r}_{N},{r}_{N}}\end{array}\end{array}\right],$$

where ${r}_{i}$ represents the $i$th ROI.

*Edge weight* For the connected edges between two nodes, the edge weight is expressed by the absolute value of the Pearson correlation coefficient between the time series of the nodes. That is, for node ${r}_{i}$ and node ${r}_{j}$, the edge weight between the two nodes is $|{\rho }_{{r}_{i}{r}_{j}}|$.

*Node feature* The node feature (or node attribute) of each node (ROI) is represented by its functional connectivity profile with the rest of the regions [8], corresponding row of the connectivity matrix, such as:

2 $${{\varvec{h}}}_{i}=\left\{{\rho }_{{r}_{i},{r}_{1}},{\rho }_{{r}_{i},{r}_{2}},\dots ,{\rho }_{{r}_{i},{r}_{N}}\right\}.$$

Based on the number of nodes N = 110, a $110\times 110$ connectivity matrix is constructed for each subject respectively, and the dimensions of node feature is 110.

### GAT2 model

**Fig. 2:**
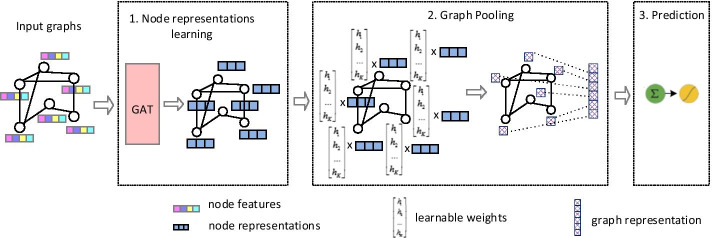
The architecture of GAT2

The architecture of the GAT2 model is illustrated in Fig. 2. The model is composed of two parts: the node representation learning part, and the pooling-and-prediction part. First, the node representation learning part learns the feature representation of the node with the graph attention networks. Then, the pooling-and-prediction part learns the graph representation based on node representation, and learns the prediction probability.

*Node representation learning* The input to the layer is a set of node features, $\mathbf{h}=\left\{{{\varvec{h}}}_{1},{{\varvec{h}}}_{2},\dots ,{{\varvec{h}}}_{N}\right\}$, ${{\varvec{h}}}_{i}\in {\mathbb{R}}^{F}$, where N is the number of nodes, F is the dimensions of node features. The graph attention layer [17] uses self-attention mechanism to aggregate the node’s 1-hop neighborhood nodes to compute the node representation. The attention coefficients are computed as follows:

3 $${a}_{ij}=softmax\left(LeakyReLU\left({{\varvec{a}}}^{{\varvec{T}}}\left[\mathrm{W}{{\varvec{h}}}_{i}\Vert \mathrm{W}{{\varvec{h}}}_{j}\right]\right)\right),$$

where ${\varvec{a}}\in {\mathbb{R}}^{2{F}^{{\prime}}}$ and the self-attention is included in ${\varvec{a}}$. Masked attention is used to introduce network structure information, and attention is only assigned to the neighbor node set ${N}_{i}$ of node $i$. The node representation generated from multi-head attention is computed as follows:

4 $${{\varvec{h}}}_{i}^{{{\prime}}}={\parallel }_{k=1}^{K}\sigma \left(\sum\limits_{j\epsilon {N}_{i}}{a}_{ij}^{k}{\mathrm{W}}^{k}{{\varvec{h}}}_{j}\right),$$

5 $${{\varvec{h}}}_{i}^{{\prime}}=\sigma \left(\frac{1}{K}\sum\limits_{k=1}^{K}\sum\limits_{j\in {N}_{i}}{a}_{ij}^{k}{\text{W}}^{k}{{\varvec{h}}}_{j}\right),$$

where the Eq. (4) uses $\parallel$ as the concatenation operation, connecting the feature representations obtained by each attention; Eq. (5) is used to obtain the node representation of the last layer by averaging the features with multiple attentions; and $\sigma =\frac{1}{1+{e}^{-x}}$.

*Graph attention pooling* For summarizing graph representation from nodes representation, we provide a sharing weight vector for every node, and the new one-dimensional representation ${P}_{i}$ of each node is obtained through function mapping, as shown in Eq. (6). Finally, we get the graph representation $\mathbf{P}=\left\{{P}_{1},{P}_{2},\dots ,{P}_{N}\right\}$ whose dimensions are equal to the number of nodes.

6 $${P}_{i}=\sigma \left({\mathrm{W}}^{p}{{\varvec{h}}}_{i}^{{\prime}}\right),$$

where $${\mathrm{W}}^{{\varvec{p}}}\in 1\times {F}^{{\prime}}$$.

*Prediction* In order to pay attention to the contribution made by each node to the final prediction result, each node representation is given a weight, and the weight calculation is shown in Eq. (7):

7 $$\mathbf{A}=softmax\left({\mathrm{W}}^{A}\mathbf{P}\right),$$

where ${\mathrm{W}}^{{\varvec{p}}}\in \mathrm{N}\times \mathrm{N}$ and $\mathbf{P}=\left\{{P}_{1},{P}_{2},\dots ,{P}_{N}\right\}$. Then, using the contribution weights, the weighted sum of the node representation is used for the prediction of the model, as shown in Eq. (8):

8 $$\mathrm{prob}=\sum\limits_{i=1}^{N}{\mathbf{A}}_{i}{P}_{i}.$$

### Interpretation methods

We use GNNExplainer [19] to interpret the trained GAT2 model, and identify the important features in GAT2 model. We use the GNNExplainer to learn a feature mask that masks out unimportant node features, i.e., where if the value of an element in feature mask matrix is closely to zero, the corresponding feature would be considered unimportant. The dimension of the feature mask matrix is 110 × 110 in this study.

### Experiments

#### Dataset and preprocessing

We used the resting-state fMRI data from 1035 subjects in the ABIDE I initiative [20] for this study. The dataset includes 505 individuals diagnosed as having ASD and 530 HC. The preprocessed resting-state fMRI data were downloaded from the Preprocessed Connectomes Project (http://preprocessed-connectomes-project.org/abide/download.html). The data were preprocessed by the Configurable Pipeline for the Analysis of Connectomes (CPAC) pipeline [24] that included the following procedure: slice timing correction, motion realignment, intensity normalization, regression of nuisance signals, band-pass filtering (0.01–0.1 Hz) and registration of fMRI images to standard anatomical space (MNI152).

#### Experimental setup

Given the above GAT2 model, we conducted experiments on the ABIDE I dataset with 1035 subjects and applied the interpretation method to explain the results.

To evaluate the performance of the proposed model, we used sensitivity, specificity, accuracy, F1 score, AUC, and Matthews correlation coefficient (MCC) as our metrics. These metrics are defined as follows:

9 $$sensitivity = \frac{TP}{{TP + FN}}$$

10 $$specificity = \frac{TN}{{TN + FP}}$$

11 $$accuracy = \frac{TP + TN}{{TP + FN + TN + FP}}$$

12 $$F1 = \frac{2 \times TP}{{2 \times TP + FP + FN}}$$

13 $$MCC = \frac{TP \times TN - FP \times FN}{{\sqrt {(TP + FP) \times (TP + FN) \times (TN + FP) \times (TN + FN)} }}$$

where true positive (TP) is defined as the number of ASD subjects that are correctly classified, false positive (FP) is the number of HC subjects that are misclassified as ASD subjects, true negative (TN) is defined as the number of HC subjects that are correctly classified, and false negative (FN) is defined as the number of ASD subjects that are misclassified as HC subjects. Sensitivity measures the proportion of correctly identified ASD subjects among all identified ASD subjects. Specificity measures the proportion of correctly identified HC subjects among all real HC subjects. AUC is defined as the area under the receiver operating characteristic curve.

#### Classification comparison models and parameters

The comparison models include (i) traditional machine learning methods: SVM, PCA + SVM, and RF; (ii) non-graph deep learning model: MLP, CNN; (iii) GCN layer based GNN models: GCN-at (1st-order), and GCN-at (Cheby); (iv) GAT layer based GNN models: GAT2, GAT-average, and GAT-fc. The comparison models and their corresponding parameters are described as follows.

*SVM* Support vector machine (SVM) model with linear kernel. SVM method is an accepted benchmark method and has been widely used to classify fMRI data for brain disorders. SVM model sets the value of parameter C to 1.0.

*PCA* + *SVM* First use principal component analysis (PCA) to reduce the dimension of feature vector and then input into the SVM model for training and classification. Using PCA to retain 99% of the feature information, the dimension is reduced to 700 dimensions, and the dimensionality-reduced vector is input into the SVM with linear kernel for training and classification. The coefficient C is set to 1.0.

*RF* Random forest (RF) is an ensemble learning method for classification. We trained RF with 300 trees, and the maximum depth of the tree is set to 30.

*MLP* The MultiLayer Perceptron (MLP) model has two fully connected layers with LeakyReLU activation function. The number of units of the two fully connected hidden layers is 64, 32 respectively. Dropout layer is added to avoid overfitting and the dropout rate is 0.5. The output layer with one neuron is followed by a sigmoid activation function. The model training uses the Adam Optimizer, the learning rate is set to 0.0005, and the loss function uses the cross-entropy loss function.

*CNN* The convolutional neural networks (CNNs) model contains three convolutional layers and two fully connected layers, the number of convolutional kernels is 32, 64, 128 respectively, the size of all kernels is 3 * 3, and the activation function uses ReLU function. The number of neurons is 1024, 2 respectively, and the activation function uses ReLU function.

*GCN-at (1st-order), GCN-at (Cheby)* In order to verify the effectiveness of GAT layer for node representation learning in the GAT2 model, we designed GCN-at (1st-order) and GCN-at (Cheby) models to classify the functional brain networks. In these two models, the GCN layer is used for training to obtain the node representation, and then the node representation is input into the same pooling-and-prediction part of GAT2 for prediction.

According to the implementation of the GCN layer proposed in [25], for the GCN-at (1st-order) model, the node representation is obtained from the GCN layer via a first-order approximation of localized spectral filters on graphs; for the GCN-at (Cheby) model, the node representation is obtained from the GCN layer via Chebyshev polynomials filter, the polynomial order is set to 3. The model contains one GCN layer, the number of units is set to 24, and the activation function uses the LeakyReLU function. The loss function uses the cross-entropy loss function.

*GAT-fc, GAT-average, GAT-learn* In order to verify the validity of the prediction part in the GAT2 model, we designed GAT-fc, GAT-learn, and GAT-average models to classify the functional brain networks.

In GAT-fc model, after obtaining the node representation vector through the GAT layer, the node representation vectors were spliced to obtain a one-dimensional vector, which is input into the fully connected layer for prediction.

The GAT-fc model contains two GAT layers, the number of attention heads is set to 5 and 3, the number of units is set to 24 and 3, respectively; the number of units of the fully connected layer is set to 64. The activation function uses LeakyReLU function. The output layer is followed by a softmax activation function. The loss function uses cross-entropy loss function.

In GAT-average model, after obtaining the node representation vector in the GAT layer, the node representation ${P}_{i}$ is mapped through the sigmoid function. Based on the average-pooling method in GCN [11], the final prediction probability of GAT-average model is obtained by averaging the information of each node, as shown in Eq. (14):

14 $$\mathrm{prob}=\frac{{\sum }_{i=1}^{N}{P}_{i}}{N}.$$

The GAT-average model contains two GAT layers, the number of attention heads is set to 5 and 3, the number of units is set to 24 and 3, respectively, and the activation function uses LeakyReLU function. The loss function uses the cross-entropy loss function.

In GAT-learn model, we use the learnable pooling method in [15] for GAT. The GAT-learn model comprises two GAT layers, one cascaded convolution-pooling blocks, and one fully-connected layer. The block generates an $N\times 11$ feature map (${Y}^{(l)}$) and an $N\times 1$ cluster assignment matrix (${S}^{T}$) in two separate paths, and combines them using pooling formulation of Eq. (15) to obtain a pooled feature map ($Y^{pool}$) of 1 * 11.

15 $$Y^{pool} = S^{T} Y^{(l)}$$

*GAT2* The model contains two GAT layers, the number of attention heads is set to 5 and 3, the number of neurons is set to 24 and 3, respectively, and the activation function uses LeakyReLU function. The node representation ${P}_{i}$ is obtained through the sigmoid function. Then the weighted sum of the node information is used for the prediction of the model. And we also set different number of GAT layers and different number of attention heads for comparing these hyper-parameters setting.

For inputs fed into non-graph learning models including SVM, PCA + SVM, RF, MLP, the upper triangle values of connectivity matrices are extracted and flattened into vectors, with the dimension of the feature vector being $\left(110\times \left(110-1\right)\right)/2=5995$. The whole connectivity matrices are used as inputs for CNN model.

All the above graph neural networks based models use Adam Optimizer for training and the learning rate is set to 0.0001. All the above deep learning models use the early stop mechanism, and the training is stopped if the test set for 15 consecutive rounds does not decrease in error rates.

#### Comparison of classification with different network construction methods

We conducted more experiments to compare the classification performance of the GAT2 model with different network construction methods.

(i) Influence of network construction via different brain atlases
  We used HO atlas [23] and Automated Anatomical Labeling (AAL) atlas [26] to divide brain regions, extracted functional connectivity features to construct brain networks, and compared the performance of classification with GAT2 model.

(ii) Influence of network sparsity
  Considering that even weak connections between nodes may record some information, so we used dense network representation for classification in the classification experiments, where the dense network is the original network without using thresholds to eliminate weak connections.

In this study, we set a threshold for the sparse brain network, and identified the influence of network sparsity. For the adjacency matrix, according to the edge weight value between nodes, only the connected edges whose edge weight value is greater than the threshold were retained. The GAT2 model was used for experimental comparison.

#### Validating GAT2 in a larger dataset

We also validated the performance of GAT2 model in a larger synthetic dataset. We constructed a graph classification dataset with 4000 graphs, where each graph had 30 nodes and the weight of each connection was randomly selected from 0 to 1. The graph dataset was divided into two categories based on the following steps: (a) 15 nodes from the graph were randomly selected; (b) the sum of the connection weights between these 15 nodes was defined as W1, the sum of the connection weights between these 15 nodes and the rest 15 nodes was defined as W2, the sum of each graph was defined as $\mathrm{W}0=\mathrm{W}1\times 2+\mathrm{W}2$, and the average value of W0 of 4000 graphs was then calculated; and (c) if W0 was larger than the average values, the category of this graph was set to Class-one, otherwise the category of the graph was set to Class-two. We also used corresponding row of the connectivity matrix to be node feature similar to the construction of brain networks described in “Construction of functional brain networks” section.

We compared the classifying performance of GAT2 model against SVM, RF, and CNN, under the similar setting with the previous experiments of ABIDE dataset. Some specific model parameters used in this experiment are as follows: The GAT2 model contained two GAT layers, the number of attention heads was set to 4 and 4, the number of neurons was set to 16 and 16, respectively; the CNN model contained three convolutional layers and two fully connected layers, the number of convolutional kernels was 16, 32, 64 respectively; the RF had 128 trees, and the maximum depth of the tree was set to 20.

#### Interpretation experiments

(i) Comparison methods
  We also used Saliency Map [21] and DeepLIFT [22] as comparative interpretation methods. Saliency Map is a typical neural network interpretation method, which is based on gradient sensitivity. To apply Saliency Map to the GAT2 model, we calculated the gradient of the model loss relative to the input features, and analyzed the features according to the gradient value. The larger the gradient value, the greater the impact the corresponding feature has on the classification. DeepLIFT is a method that can decompose the output prediction of a neural network on a specific input by back propagating the contributions of all neurons in the network to each feature of the input.

We explored the impact of features on classifying functional brain networks of the ASD individuals. The sample feature dimension of the input model is N × F, in which N represents the number of nodes, and F represents the node feature dimension. As described in “GAT2 model” section, the constructed network has N = 110 network nodes and F = 110 features of each node. The steps of obtaining the characteristic gradient value are as follows: (a) for the test samples, the gradient of the model loss relative to the input features was calculated to obtain the gradient value of each feature; (b) for each feature, the average value of the gradient across all samples was identified and the absolute value of them was calculated.

(ii) Interpretation experiments
  We applied Saliency Map, DeepLIFT, and GNNExplainer to interpret the trained GAT2 model, and estimated the classification performance impact of GAT2 models by the feature perturbation. We then compared the change of GAT2’s prediction when modifying the same number of features to compare the quality of the two interpretation methods.

We hacked the model by setting value of the nodal feature in instance x to zero, and observe the changes of prediction of GAT2 in one-fold data from the above fivefold cross-validation data division. We used metrics including sensitivity, specificity, accuracy, the change of prediction probability (CPP) which is the absolute change of probability of classifying $\mathrm{x}$ as a positive instance, the number of label-changed instance (NLCI) which is the number of instances whose predicted label changes after being hacked.

## Results

### Classification results

#### Results of comparison models

The classification results of each model are shown in Table 1. After randomly performing fivefold cross-validation data division, in each round of experiments, one-fold data were used for testing while other fourfold data were used for training the model. A specialized computer with i7-6700 K CPU, 64 GB RAM, and a NVIDIA GTX 1080 Ti GPU was used to train the models. For training GAT2 model, average number of epochs was 232, training batch size was 10, and the average training time was 329.9 s.

**Table 1:** Classification performance of each model (mean $\pm$ std)

Model	Accuracy	Sensitivity	Specificity	F1	AUC	MCC
SVM	0.6618 ± 0.0110	0.6515 ± 0.0413	0.6717 ± 0.0218	0.6521 ± 0.0211	0.7170 ± 0.0188	0.3238 ± 0.0230
PCA + SVM	0.6686 ± 0.0195	0.6554 ± 0.0561	0.6811 ± 0.0334	0.6576 ± 0.0300	0.7184 ± 0.0156	0.2793 ± 0.0339
RF	0.6599 ± 0.0309	0.5921 ± 0.0309	**0.7245 ± 0.0324**	0.6295 ± 0.0330	0.7153 ± 0.0325	0.2978 ± 0.0768
MLP	0.6754 ± 0.0309	0.6634 ± 0.0401	0.6868 ± 0.0601	0.6660 ± 0.0297	**0.7535 ± 0.0297**	0.2899 ± 0.0612
CNN	0.6550 ± 0.0312	0.6316 ± 0.0466	0.6774 ± 0.0345	0.6407 ± 0.0364	0.7111 ± 0.0314	0.3098 ± 0.0615
GCN-at (1st-order)	0.5971 ± 0.0460	0.6059 ± 0.0398	0.5887 ± 0.0619	0.5951 ± 0.0417	0.6537 ± 0.0503	0.2775 ± 0.0645
GCN-at (Cheby)	0.6357 ± 0.0217	0.6812 ± 0.0558	0.5925 ± 0.0558	0.6452 ± 0.0262	0.6926 ± 0.0368	0.2975 ± 0.0600
GAT-fc	0.6184 ± 0.0332	0.7089 ± 0.0507	0.5321 ± 0.0927	0.6445 ± 0.0209	0.6547 ± 0.0426	0.3155 ± 0.0713
GAT-average	0.6734 ± 0.0354	0.7386 ± 0.0270	0.6113 ± 0.0801	0.6889 ± 0.0226	0.7361 ± 0.0321	0.3237 ± 0.0621
GAT-learn	0.5845 ± 0.0371	0.6000 ± 0.1765	0.5698 ± 0.1473	0.5732 ± 0.0844	0.5849 ± 0.0385	0.1798 ± 0.0821
GAT2	**0.6802 ± 0.0269**	**0.7406 ± 0.0408**	0.6226 ± 0.0534	**0.6931 ± 0.0248**	0.7358 ± 0.0373	**0.3426 ± 0.0628**

The bold means it is the best result for each metric (column of the table)

The GAT2 model achieved the best results in accuracy, sensitivity, F1 score, and MCC indicators using fivefold cross-validation, with the accuracy of 68.02%, sensitivity of 74.06%, F1 score of 69.31%, and MCC of 0.3426.

From Table 1, we could find that the deep learning models (MLP and GAT2) achieved better performance than the traditional machine learning methods (SVM, PCA + SVM). The MLP model achieved the highest AUC value of 0.7535. The accuracy, sensitivity, F1 score, and MCC of the GAT2 model were higher than the MLP model, and the total classification performance was slightly better than the MLP model.

Compared with GCN layer based graph models, the classification performance of the GAT2 model (with GAT layers) was better than GCN-at (1st-order) and GCN-at (Cheby) with GCN layers.

Compared the GAT layer based models, GAT2 model achieved the best results. The classification performance of the three was GAT2 > GAT-average > GAT-fc > GAT-learn. In GAT-learn, there are two separate paths of neural networks to learn the pooling strategy, and the worst performance of this model may be due to the complex structure which makes it easy to overfit for this dataset. In GAT-fc, the node representation output from the GAT layer was flattened into a one-dimensional vector, and then entered to the fully connected layer for training and classification. The bad performance of GAT-fc may be due to the direct splicing of the node representation, which lost the information learned by each node. GAT-average, which retains the information of each node on average, does not consider that different nodes may contribute differently to the prediction results, so the classification effect was not as good as GAT2; GAT2 uses a weighted layer to learn each node representation, the information of each node was retained for final prediction, and the performance was significantly improved.

In summary, the proposed GAT2 model achieves the best results compared to other ten models, including SVM, PCA + SVM, RF, MLP, CNN, GCN-at (1st-order), GCN-at (Cheby), GAT-fc, GAT-average, and GAT-learn.

#### Results of GAT2 with different neural network structures

The results of GAT2 with different neural network structures are shown in Table 2. We compared different number of attention layers, and the number of attention multi-head for each layer.

**Table 2:** Performance of GAT2 with different neural network structures (mean $\pm$ std)

Number of layers	Number of multi-head for each layer	Accuracy	Sensitivity	Specificity	F1	AUC	MCC
1	5	0.6696 ± 0.0332	0.6416 ± 0.0440	**0.6962 ± 0.0307**	0.6541 ± 0.0378	0.7251 ± 0.0388	0.3385 ± 0.0668
3	5, 5, 3	0.6415 ± 0.0422	0.6435 ± 0.1286	0.6396 ± 0.0629	0.6312 ± 0.0629	0.7145 ± 0.0480	0.2923 ± 0.0796
2	5, 5	0.6676 ± 0.0409	0.6812 ± 0.0706	0.6547 ± 0.0935	0.6660 ± 0.0404	0.7261 ± 0.0384	0.3390 ± 0.0787
2	3, 3	0.6599 ± 0.0371	0.6753 ± 0.0413	0.6453 ± 0.0642	0.6597 ± 0.0337	0.7178 ± 0.0529	0.3214 ± 0.0731
2	5, 3	**0.6802 ± 0.0269**	**0.7406 ± 0.0408**	0.6226 ± 0.0534	**0.6931 ± 0.0248**	**0.7358 ± 0.0373**	**0.3426 ± 0.0628**

The bold means it is the best result for each metric (column of the table)

#### Results of classification with different network construction methods

(i) Influence of network construction via different brain atlases
  The classification results of using AAL and HO atlas are shown in Table 3. Compared with the AAL atlas, using the HO atlas for construction of the brain network, with the same model, the accuracy was increased by about 5%, the sensitivity was increased by about 2%, and the F1 value was increased by about 4%. All evaluation metrics have been significantly improved when using the HO atlas.

**Table 3:** Classification performance on different brain atlases (mean $\pm$ std)

Atlas	Accuracy	Sensitivity	Specificity	F1	AUC
AAL	0.6300 ± 0.0428	0.7188 ± 0.0298	0.5453 ± 0.0988	0.6556 ± 0.0236	0.6763 ± 0.0499
HO	**0.6802 ± 0.0269**	**0.7406 ± 0.0408**	**0.6226 ± 0.0534**	**0.6931 ± 0.0248**	**0.7358 ± 0.0373**

The bold means it is the best result for each metric (column of the table)
(ii) Influence of brain network sparsity
  The classification results of using different network sparsity are shown in Table 4. The number of edges and sparsity of the brain network are shown with different threshold for edge weight. As can be seen from the table, when the network became more and more sparser, the accuracy, specificity and F1 value of the model continued to decline. For the two metrics of sensitivity and AUC value, as a whole, as the network became sparser, the value also showed a downward trend. When the threshold was greater than 0.3, the eliminated node connection edges increased, and each index decreased by a large extent. Even if the threshold value was 0.1, the classification accuracy of the model still decreased. It indicates that retaining the weak connection information of the network can enable the node to learn more information from neighboring nodes in this model, which allowed the model to achieve better classification performance.

**Table 4:** Classification performance on networks with different sparsity (mean $\pm$ std)

Threshold	Number of edges	Sparisty	Accuracy	Sensitivity	Specificity	F1	AUC
0.1	10,056	0.1689	0.6686 ± 0.0344	0.7287 ± 0.0358	0.6113 ± 0.0790	0.6826 ± 0.0236	**0.7396 ± 0.0317**
0.2	7976	0.3408	0.6512 ± 0.0604	0.7327 ± 0.0360	0.5736 ± 0.1110	0.6738 ± 0.0433	0.7165 ± 0.0603
0.3	5877	0.5142	0.6377 ± 0.0684	0.7149 ± 0.0449	0.5642 ± 0.1255	0.6600 ± 0.0467	0.7005 ± 0.0648
0.4	3933	0.6750	0.6232 ± 0.0693	0.7129 ± 0.0429	0.5377 ± 0.1303	0.6506 ± 0.0460	0.6878 ± 0.0681
0.5	2330	0.8074	0.6145 ± 0.0516	0.6594 ± 0.0409	0.5717 ± 0.0992	0.6263 ± 0.0370	0.6831 ± 0.0542
Dense network	12,100	0	**0.6802 ± 0.0269**	**0.7406 ± 0.0408**	**0.6226 ± 0.0534**	**0.6931 ± 0.0248**	0.7358 ± 0.0373

The bold means it is the best result for each metric (column of the table)

#### Results of validating GAT2 in the larger dataset

The classification results in the larger constructed graph dataset are shown in Table 5. The GAT2 model achieved the best results in accuracy, sensitivity, F1 score, AUC, and MCC indicators using fivefold cross-validation, with the accuracy of 95.18%, sensitivity of 95.68%, specificity of 94.66%, F1 score of 95.26%, AUC of 95.17%, and MCC of 99.78%.

**Table 5:** Classification performance in the larger graph dataset (mean $\pm$ std)

Model	Accuracy	Sensitivity	Specificity	F1	AUC	MCC
SVM	0.9242 ± 0.0114	0.9235 ± 0.0133	0.9249 ± 0.0129	0.9247 ± 0.0113	0.9242 ± 0.0114	0.8485 ± 0.0228
RF	0.5975 ± 0.0127	0.6367 ± 0.0210	0.5541 ± 0.0094	0.6133 ± 0.0151	0.5954 ± 0.0127	0.1916 ± 0.0257
CNN	0.5917 ± 0.0172	0.6714 ± 0.0383	0.5160 ± 0.0394	0.5559 ± 0.1888	0.5911 ± 0.0017	0.3018 ± 0.0259
GAT2	**0.9518 ± 0.0121**	**0.9568 ± 0.0344**	**0.9466 ± 0.0059**	**0.9526 ± 0.0099**	**0.9517 ± 0.0123**	**0.9978 ± 0.0006**

The bold means it is the best result for each metric (column of the table)

### Explanation experiments

**Fig. 3:**
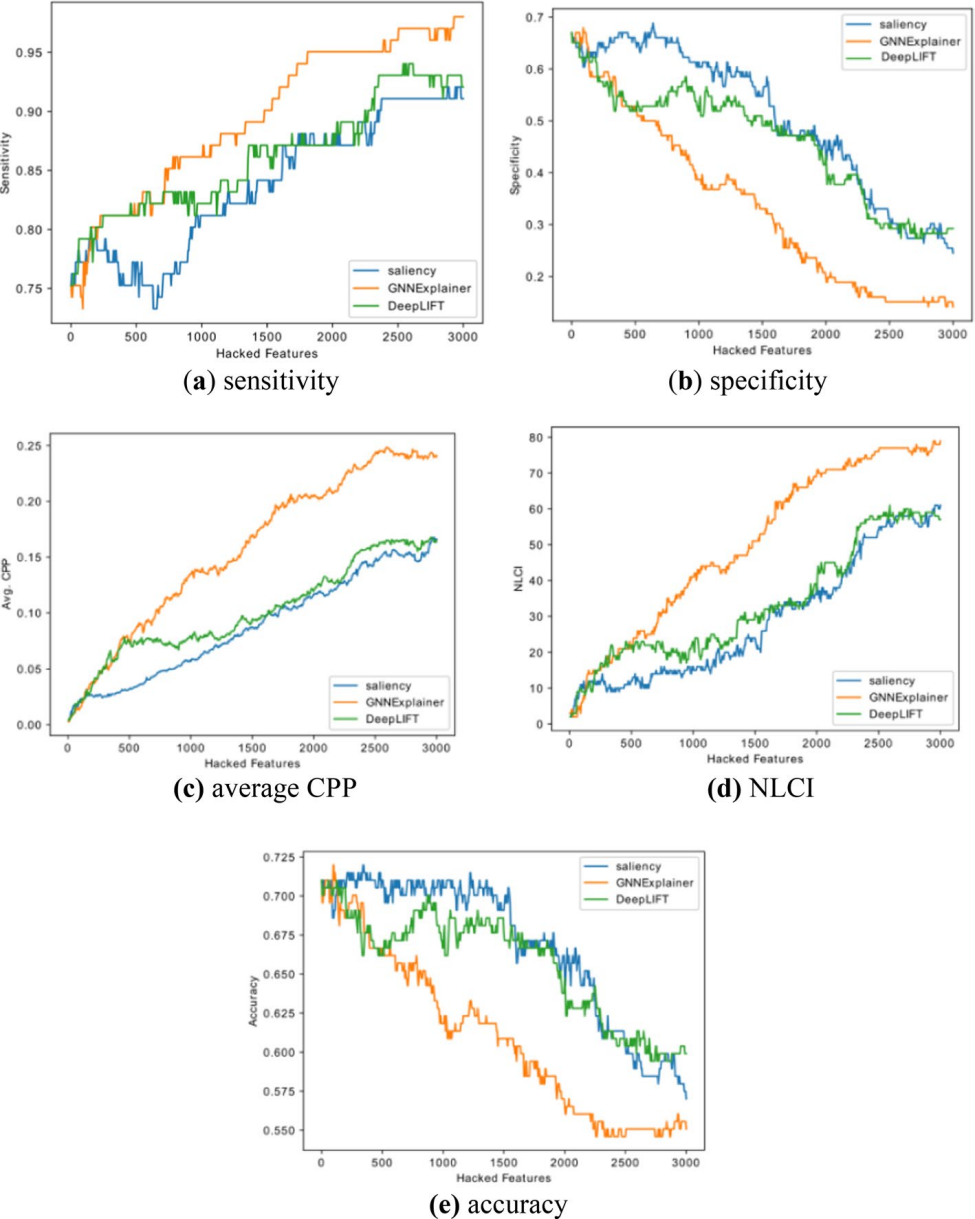
The performance of top features on Saliency Map and GNNExplaine

The results of using Saliency Map, DeepLIFT, and GNNExplainer methods for GAT2 model on the ABIDE dataset are shown in Fig. 3. It’s shown that the average CPP and NLCI of GNNExplainer were higher than Saliency Map. And GNNExplainer achieved a bigger change of prediction in sensitivity, specificity and accuracy. It demonstrated that GNNExplainer performed better than Saliency Map when interpreting GAT2 model.

We further analyzed the impact of top features of GNNExplainer method with Fig. 3, and it could be found that there is a significant impact on sensitivity, specificity, NLCI, and accuracy when hacking the top features. As seen in Fig. 3e, we could find that the decline curve of the accuracy had two stages, the first stage dropping faster, and the latter stage dropping more slowly. In the first stage, the accuracy would drop to 0.6470 when hacking the top 605 features; in the latter stage, the accuracy would drop to 0.5603 when hacking the top 2115 features. It indicates that these 605 features have contributed more to the classification of ASD from HC, and the rest of 1510 features, while also having significant impacts on the classification in GAT2 model, do not contribute as much as these 605 features.

We selected the top 10 connections (rsFCs) as shown in Table 6. We computed the mean value of each rsFC of the ASD group and the HC group, respectively, as well as the mean difference of two groups. An independent two-sample t test was run on the means of the rsFC elements of two groups.

**Table 6:** Analyses of 10 rsFCs

Connection ID	ROI number	Regions	ASD mean connection	HC mean connection	Mean difference	*p* value
1	31	Right Superior Parietal Lobule	0.5816	0.5580	0.0237	0.3161
	33	Right Supramarginal Gyrus; posterior division				
2	31	Right Superior Parietal Lobule	0.3633	0.3536	0.0097	0.7276
	38	Right Frontal Medial Cortex				
3	11	Right Hippocampus	0.2543	0.2191	0.0353	0.2224
	39	Right Juxtapositional Lobule Cortex (formerly Supplementary Motor Cortex)				
4	11	Right Hippocampus	0.3320	0.2586	0.0734	0.0097**
	38	Right Frontal Medial Cortex				
5	62	Left Frontal Pole	0.3600	0.4201	− 0.0600	0.0109**
	19	Right Inferior Frontal Gyrus; pars opercularis				
6	62	Left Frontal Pole	0.0138	− 0.0081	0.0219	0.4854
	51	Right Temporal Fusiform Cortex; posterior division				
7	62	Left Frontal Pole	0.4269	0.4778	− 0.0509	0.0375**
	68	Left Precentral Gyrus				
8	31	Right Superior Parietal Lobule	0.3830	0.3622	0.0208	0.4224
	5	Left Amygdala				
9	62	Left Frontal Pole	− 0.0692	− 0.1137	0.0445	0.1646
	94	Left Frontal Orbital Cortex				
10	62	Left Frontal Pole	0.4016	0.3743	0.0273	0.3308
	42	Right Cingulate Gyrus; anterior division				

**p < 0.05

In addition, we also used GNNExplainer to explain the GAT2 model of synthetic graph dataset, and the top 10 connections are shown in Table 7. The mean value of each connection of the Class_one group and the Class_two group, the mean difference of two groups, and the P values were computed similarly as in Table 6.

**Table 7:** Analyses of 10 connections in synthetic graph dataset

Connection ID	Node ID	Class_one mean connection	Class_two mean connection	Mean difference	*p* value
1	16	0.5052	0.4916	0.0135	0.0362**
	5				
2	13	0.5160	0.4962	0.0197	0.0023**
	5				
3	4	0.5079	0.4904	0.0175	0.0073**
	1				
4	15	0.5061	0.4995	0.0066	0.3090
	5				
5	22	0.5068	0.4978	0.0091	0.1554
	5				
6	27	0.5144	0.4864	0.0272	0.00002**
	30				
7	14	0.5109	0.4837	0.0272	0.00002**
	5				
8	28	0.5121	0.4860	0.0260	0.0039**
	28				
9	27	0.5064	0.4900	0.0164	0.0109**
	1				
10	26	0.5059	0.4846	0.0219	0.0011**
	19				

**p < 0.05

## Discussion

The superior performance of GAT2 model in classifying functional brain networks stems from two key aspects of the graph neural networks: graph attention learning layers for node representation, and attention learning in graph pooling. Graph attention layers are able to attend to neighborhoods' features, and enable specifying different weights for different nodes in a neighborhood. Compared with GCN layer based graph models, such as GCN-at (1st-order) and GCN-at (Cheby), GAT layer based graph models (GAT2 and GAT-average) yielded higher AUC score in the experiments. And the attention learning for graph pooling, which uses learnable parameters to summarize graph representation with a concise strategy, enhances the representation ability of graph. Compared with other pooling methods, such as in GAT-fc, and GAT-average models, the proposed graph attention pooling in GAT2 model achieves higher accuracy, sensitivity, specificity, and F1 score. To further demonstrate the utility and power of GAT2 model, we used more data to validate the GAT2 model in a larger graph dataset with 4000 samples, and the results showed that the performance of GAT2 model has been significantly better than the other comparison models.

For the construction of the brain network, we found that compared with the AAL atlas, GAT2 using HO atlas can capture the functional differences between the brain networks of ASD and HC in this dataset. It may be that numerical values of the underlying network metrics and the relation between nodal properties and region size were dependent on the atlas used [27, 28], and compared with the AAL atlas, GAT2 using HO atlas can capture the functional differences between the brain networks of ASD individuals and HC in this dataset. Compared with sparse networks obtained by threshold, the dense network with weak connection information could enable the node to learn more information from neighboring nodes in GAT2 model.

For model interpretation, GNNExplainer performed better than Saliency Map and DeepLIFT when interpreting GAT2 model. We think that GNNExplainer is more powerful for interpreting the GAT2 model than Saliency and DeepLIFT. This is because the weights and attentions of features in the trained GAT2 model are similar, and the gradient values of features are similar, making it difficult to find the salient features by comparing gradient values with Saliency Map or DeepLIFT, while it is easier for GNNExplainer to learn the feature masks to obtain the salient features.

For interpreting results of the model from functional brain networks, as shown in the Table 6, the top 10 connections (rsFCs) involved 12 ROIs (brain regions), and among these 10 rsFCs, 3 rsFCs (the connection 4, 5, and 7) were statistically significant (*p* < 0.05) between the ASD and HC groups. The connection 1, 2, and 8 were associated with the Right Superior Parietal Lobule. In the ASD group, the Right Superior Parietal Lobule was strongly correlated with the Right Supramarginal Gyrus posterior division, and relatively weakly correlated with the Right Frontal Medial Cortex and the Left Amygdala. Such abormal rsFC connection patterns may result from increased or decreased key ROI/brain regions in information processing, as previous studies indicated. For example, decreased activation of the Right Superior Parietal Lobule has been observed in individuals with ASD during learning [29]. Further, the connection 3 and 4 are associated with the Right Hippocampus. The connection of the Right Hippocampus with the Right Frontal Medial Cortex was stronger in the ASD group than in the HC group. It has been found that children with ASD show reduced working-memory-related activations in the right hippocampus [30]. The connection 5, 6, 7, 9, and 10 are all associated with the Left Frontal Pole. The connections of the Left Frontal Pole with the Right Inferior Frontal Gyrus pars opercularis, and the Left Frontal Pole with the Left Precentral Gyrus, were weaker in the ASD group than in the HC group. Differences have been observed in Left Frontal Pole when studying the longitudinal changes of cortical thickness in autism and typical development [31], along with greater activation of Left Frontal Pole in the ASD group during reward anticipation and outcomes for monetary and social rewards [32]. Finally, it should be noted that among these 10 connections in Tables 6 and 7 connections are not statistically significant between the ASD and HC groups. That may be because the sample size of the groups was not large enough to reveal the statistical power [33]. Nevertheless, they had contributed to the classification of ASD and HC in the GAT2 model found by the GNN explanation method.

The proposed GAT-LI method has the potential in assisting future diagnoses of brain neurological disorders such as ASD, in addition to understanding the neural bases of ASD, since the two-stage method could learn an accurate GNN model for graph data and interpret how specific decisions of these graph models are made by feature importance. Besides, GAT-LI could be generalized to the classification and interpretation tasks of graph data from other biomedical fields.

There are two limitations in the current work. First, the brain network dataset is limited to the ASD classification task. It would be important to see whether the proposed GAT-LI excels in classifying and interpreting other brain network data. Second, our brain network dataset is limited to 1035 participants, although we used the larger synthetic dataset to validate the utility of GAT2 model. Future studies should rely on large-scale real data of both typically developing individuals and individuals with neuropsychological disorders.

## Conclusions

This paper proposes a graph attention network based Learning and Interpreting method, namely GAT-LI, which uses a graph attention network model to learn to classify functional brain networks of ASD versus HC, and uses GNNExplainer to interpret the learned graph model. For the learning model, we proposed GAT2, which uses GAT layers to learn node representations and a novel attention pooling layer to obtain the functional brain network representation for classification. The results of our experiments showed that GAT2 model outperformed the other comparison models for classifying ASD from HC in the ABIDE database. We also compared the classification performance of our model in different brain networks, including the brain networks constructed with different brain atlases, and the sparsity of brain networks on different connection thresholds. We also further constructed a larger synthetic dataset to conduct more experiments to demonstrate the utility and power of GAT2 model. Finally, we used GNNExplainer to interpret the GAT2 model, and identified the significant features in classifying brain networks of ASD individuals from HC. Future work should focus on the accuracy and application of the GAT-LI method in analyzing other large-scale brain network data from both normal and disordered populations.

## Data Availability

The datasets analyzed during the current study are available in the ABIDE Preprocessed Connectomes Project website of http://preprocessed-connectomes-project.org/abide/download.html. The source codes of GAT-LI are publicly available at the project website of https://github.com/largeapp/gat-li.

## Abbreviations

AAL: Automated Anatomical Labeling
ABIDE I: Autism Brain Imaging Data Exchange I
ASD: Autism spectrum disorder
AUC: Area under the receiver operating characteristic curve
CNN: Convolutional neural network
CPAC: Configurable Pipeline for the Analysis of Connectomes
CPP: Change of prediction probability
DHP: Dense hierarchical pooling
fMRI: Functional Magnetic Resonance Imaging
FN: False negative
FP: False positive
GAT: Graph attention network
GCN: Graph convolutional networks
GNN: Graph neural networks
HC: Healthy controls
HO: Harvard Oxford
MLP: MultiLayer Perceptron
NLCI: Number of label-changed instance
PCA: Principal component analysis
RF: Random forest
ROIs: Regions of interests
rsFC: Resting-state functional connectivity
SVM: Support vector machine
TN: True negative
TP: True positive

## References

[1] Khosla M, Jamison K, Ngo GH, Kuceyeski A, Sabuncu MR (2019). Machine learning in resting-state fMRI analysis. Magn Reson Imaging.

[2] Sólon A, Rosa A, Craddock RC, Buchweitz A, Meneguzzi F (2018). Identification of autism spectrum disorder using deep learning and the ABIDE dataset. NeuroImage Clin.

[3] Guo X, Dominick KC, Minai AA, Li H, Erickson CA, Lu LJ (2017). Diagnosing autism spectrum disorder from brain resting-state functional connectivity patterns using a deep neural network with a novel feature selection method. Front Neurosci.

[4] Eslami T, Mirjalili V, Fong A, Laird A, Saeed F (2019). ASD-DiagNet: a hybrid learning approach for detection of autism spectrum disorder using fMRI data. Front Neuroinform.

[5] Hu J, Cao L, Li T, Liao B, Dong S, Li P (2020). Interpretable learning approaches in resting-state functional connectivity analysis: the case of autism spectrum disorder. Comput Math Methods Med.

[6] Li X, Dvornek NC, Zhuang J, Ventola P, Duncan JS. Brain biomarker interpretation in ASD using deep learning and Fmri. In: International conference on medical image computing and computer-assisted intervention. Cham: Springer; 2018. p. 206–214. 10.1007/978-3-030-00931-1_24 PMC7519581 32984865

[7] Li X, Dvornek NC, Zhou Y, Zhuang J, Ventola P, Duncan JS. Efficient interpretation of deep learning models using graph structure and cooperative game theory: application to asd biomarker discovery. In: International conference on information processing in medical imaging. Cham: Springer; 2019. p. 718–730. 10.1007/978-3-030-20351-1_56 PMC7519580 32982121

[8] Ktena SI, Parisot S, Ferrante E, Rajchl M, Lee M, Glocker B, Rueckert D (2018). Metric learning with spectral graph convolutions on brain connectivity networks. Neuroimage.

[9] Ma G, Ahmed NK, Willke T, Sengupta D, Cole MW, Turk-Browne NB, Yu PS. Similarity learning with higher-order graph convolutions for brain network analysis. arXiv preprint arXiv:1811.02662 (2018).

[10] Zhang X, Chou J, Wang F. Integrative analysis of patient health records and neuroimages via memory-based graph convolutional network. In: 2018 IEEE International conference on data mining (ICDM). IEEE; 2018. p. 767–776.

[11] Arslan S, Ktena SI, Glocker B, Rueckert D, Stoyanov D, Taylor Z, Ferrante E, Dalca AV (2018). Graph saliency maps through spectral convolutional networks: application to sex classification with brain connectivity. Graphs in biomedical image analysis and integrating medical imaging and non-imaging modalities.

[12] Yang H, Li X, Wu Y, Li S, Lu S, Duncan JS, Gee JC, Gu S. Interpretable multimodality embedding of cerebral cortex using attention graph network for identifying bipolar disorder. In: International conference on medical image computing and computer-assisted intervention. Cham: Springer; 2019. p. 799–807.

[13] Zhou J, Cui G, Zhang Z, Yang C, Liu Z, Wang L, Li C, Sun M. Graph neural networks: a review of methods and applications. arXiv preprint arXiv:1812.08434 (2018).

[14] Wu Z, Pan S, Chen F, Long G, Zhang C, Philip SY (2020). A comprehensive survey on graph neural networks. IEEE Trans Neural Netw Learn Syst.

[15] Gopinath K, Desrosiers C, Lombaert H. Learnable pooling in graph convolution networks for brain surface analysis. IEEE Trans Pattern Anal Mach Intell (2020). 10.1109/TPAMI.2020.3028391 33006927

[16] Ying Z, You J, Morris C, Ren X, Hamilton W, Leskovec J. Hierarchical graph representation learning with differentiable pooling. In: Advances in neural information processing systems (NeurIPS 2018); 2018. p. 4800–4810.

[17] Veličković P, Cucurull G, Casanova A, Romero A, Lio P, Bengio Y. Graph attention networks. arXiv preprint arXiv:1710.10903 (2017).

[18] Lee J, Lee I, Kang J. Self-attention graph pooling. arXiv preprint arXiv:1904.08082 (2019).

[19] Ying Z, Bourgeois D, You J, Zitnik M, Leskovec J. Gnnexplainer: generating explanations for graph neural networks. In: Advances in neural information processing systems (NeurIPS 2019); 2019. p. 9240–9251. PMC7138248 32265580

[20] Di Martino A, Yan C-G, Li Q, Denio E, Castellanos FX, Alaerts K, Anderson JS, Assaf M, Bookheimer SY, Dapretto M (2014). The autism brain imaging data exchange: towards a large-scale evaluation of the intrinsic brain architecture in autism. Mol Psychiatry.

[21] Simonyan K, Vedaldi A, Zisserman A. Deep inside convolutional networks: visualising image classification models and saliency maps. arXiv preprint arXiv:1312.6034 (2013).

[22] Shrikumar A, Greenside P, Kundaje A. Learning important features through propagating activation differences. In: International conference on machine learning. PMLR; 2017. p. 3145–3153.

[23] Desikan RS, Ségonne F, Fischl B, Quinn BT, Dickerson BC, Blacker D, Buckner RL, Dale AM, Maguire RP, Hyman BT (2006). An automated labeling system for subdividing the human cerebral cortex on MRI scans into gyral based regions of interest. Neuroimage.

[24] Craddock C, Sikka S, Cheung B, Khanuja R, Ghosh SS, Yan C, Li Q, Lurie D, Vogelstein J, Burns R (2013). Towards automated analysis of connectomes: the configurable pipeline for the analysis of connectomes (C-PAC). Front Neuroinform.

[25] Kipf TN, Welling M. Semi-supervised classification with graph convolutional networks. arXiv preprint arXiv:1609.02907 (2016).

[26] Tzourio-Mazoyer N, Landeau B, Papathanassiou D, Crivello F, Etard O, Delcroix N, Mazoyer B, Joliot M (2002). Automated anatomical labeling of activations in SPM using a macroscopic anatomical parcellation of the MNI MRI single-subject brain. Neuroimage.

[27] de Reus MA, Van den Heuvel MP (2013). The parcellation-based connectome: limitations and extensions. Neuroimage.

[28] Wang J, Wang L, Zang Y, Yang H, Tang H, Gong Q, Chen Z, Zhu C, He Y (2009). Parcellation-dependent small-world brain functional networks: a resting-state fMRI study. Hum Brain Mapp.

[29] Travers BG, Kana RK, Klinger LG, Klein CL, Klinger MR (2015). Motor learning in individuals with autism spectrum disorder: activation in superior parietal lobule related to learning and repetitive behaviors. Autism Res.

[30] Urbain CM, Pang EW, Taylor MJ (2015). Atypical spatiotemporal signatures of working memory brain processes in autism. Transl Psychiatry.

[31] Zielinski BA, Prigge MBD, Nielsen JA (2014). Longitudinal changes in cortical thickness in autism and typical development. Brain.

[32] Dichter GS, Richey JA, Rittenberg AM (2012). Reward circuitry function in autism during face anticipation and outcomes. J Autism Dev Disord.

[33] Demšar J (2006). Statistical comparisons of classifiers over multiple data sets. J Mach Learn Res.

